# A Multistep High-Content Screening Approach to Identify Novel Functionally Relevant Target Genes in Pancreatic Cancer

**DOI:** 10.1371/journal.pone.0122946

**Published:** 2015-04-07

**Authors:** Malte Buchholz, Tatjana Honstein, Sandra Kirchhoff, Ramona Kreider, Harald Schmidt, Bence Sipos, Thomas M. Gress

**Affiliations:** 1 Clinic for Gastroenterology, Endocrinology, Metabolism and Infectiology, Philipps-University Marburg, Marburg, Germany; 2 Department of Pathology and Neuropathology, University Hospital Tübingen, Tübingen, Germany; Georgia Regents University, UNITED STATES

## Abstract

In order to foster the systematic identification of novel genes with important functional roles in pancreatic cancer, we have devised a multi-stage screening strategy to provide a rational basis for the selection of highly relevant novel candidate genes based on the results of functional high-content analyses. The workflow comprised three consecutive stages: 1) serial gene expression profiling analyses of primary human pancreatic tissues as well as a number of *in vivo* and *in vitro* models of tumor-relevant characteristics in order to identify genes with conspicuous expression patterns; 2) use of ‘reverse transfection array’ technology for large-scale parallelized functional analyses of potential candidate genes in cell-based assays; and 3) selection of individual candidate genes for further in-depth examination of their cellular roles. A total of 14 genes, among them 8 from “druggable” gene families, were classified as high priority candidates for individual functional characterization. As an example to demonstrate the validity of the approach, comprehensive functional data on candidate gene ADRBK1/GRK2, which has previously not been implicated in pancreatic cancer, is presented.

## INTRODUCTION

PDAC is a fatal disease that has a five-year survival rate below of 6%, which is the lowest for all solid tumours [1]. Early symptoms are rare and uncharacteristic, so that only 8% of PDAC cases will be in a localised and resectable stage at the time of diagnosis, whereas the majority of patients will be diagnosed in an advanced, regional (27%) or distant (53%) stage. Since advanced PDAC is inherently resistant to most available therapies, most patients die four to six months after diagnosis [1].

Patients with metastatic disease receive systemic palliative chemotherapy, with gemcitabine being the standard of care [2]. Numerous futile trials have been conducted to improve the outcome in patients with metastatic disease by using combinations with a gemcitabine-backbone. Only recently, new combination chemotherapy regimens, such as FOLFIRNOX [3] achieved a significant survival benefit, though at the cost of significantly enhanced toxicity. Multiple trials of novel targeted therapies failed to demonstrate superiority over gemcitabine alone [4], with the tyrosin kinase inhibitor erlotinib being the only targeted agent to show a minor but significant survival benefit of 14 days [5]. Novel approaches to improve drug delivery, such as albumin-bound paclitaxel [6], have been developed and show promising results in first trials, but will ave to be substantiated. There is thus a continuing and pressing need to identify novel concepts and targets which may provide new avenues to battle this disease.

As for many other diseases, large-scale genomic, transcriptomic and, to a somewhat lesser degree, proteomic analyses have been instrumental in establishing comprehensive catalogues of molecules that are altered in their structure and/or abundance in pancreatic tumours (e.g. [7–21]. Far less developed are concepts and methods to interrogate gene functions on a large scale in order to differentiate “driver” alterations, which directly contribute to malignant transformation and tumour progression, from “passenger” alterations, which have minimal or no influence on tumour biology. As a consequence, examples of successful translation of knowledge generated from “omics” approaches into novel clinical concepts and applications are few and scarce.

We describe here a multistage strategy designed to circumvent this problem by providing a rational basis for the selection of highly relevant novel candidate genes based on the results of functional high-content analyses. To this end, the workflow of the study comprised three different stages: 1) serial gene expression profiling analyses of primary human pancreatic tissues as well as a number of *in vivo* and *in vitro* models of pancreas development, cell differentiation, invasion, metastasis, apoptosis resistance etc. using custom arrays of focussed cDNA collections in order to identify genes with conspicuous expression patterns; 2) use of ‘reverse transfection array’ technology [22,23] for large-scale parallelized functional analyses of candidate genes selected from stage 1 in cell-based assays performed in microscope slide array format; and 3) selection of individual candidate genes showing relevant functional effects in parallelized assays for further in-depth examination of their cellular roles. As an example to demonstrate the validity of this approach, comprehensive data on previously unknown functions of one candidate gene selected through this process (ADRBK1/GRK2) in pancreatic cancer cells is presented.

**Fig 1:**
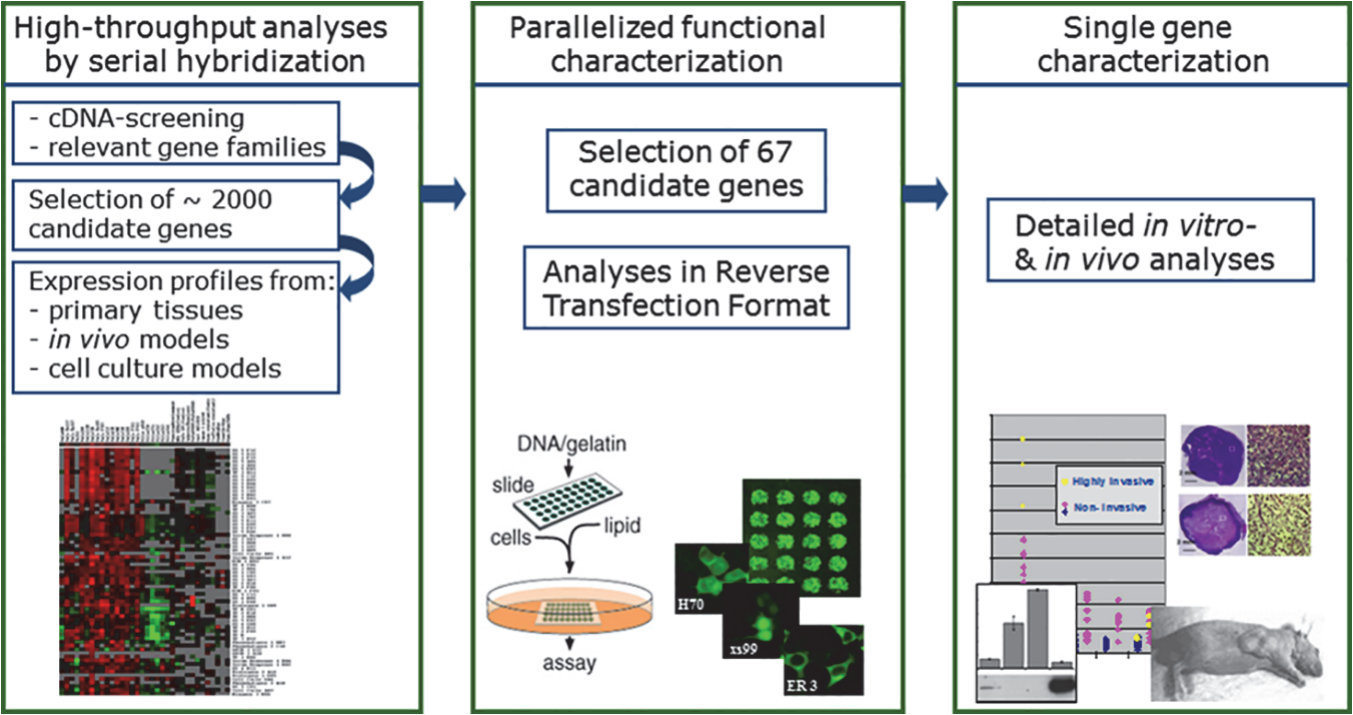
Schematic representation of the workflow in the different stages of the screening and functional characterization process.

The complete workflow of the multistep gene selection/characterization process is schematically outlined in Fig 1.

## MATERIALS AND METHODS

### cDNA array production and hybridization

Production of cDNA arrays, extraction and radioactive labeling of total RNA, as well as hybridization and quantification of hybridization signals were performed as previously described [24,25]. In brief, cDNAs were PCR amplified and arrayed in duplicate onto nylon membranes using robotic devices.

Total RNA reverse transcribed and radiactively labeled with 33P-dATP using the StripEZ-RT Kit (Ambion) and hybridized over night to nylon membrane arrays in ULTRArray hybridization buffer (Ambion) at 50°C. Radioactive signals were detected using a STORM phosphor imaging system (Amersham Biosciences, Feiburg, Germany) and quantified with the ArrayVision software (InterFocus, Haverhill, Great Britain). Signal intensities were normalized to the mean signal intensity of all features on an individual array.

Genes were defined as differentially expressed between two sets of samples if: (1) the mean normalised expression value exceeded 0.5 in at least one of the two sample sets, (2) the difference between the mean normalised expression values was at least twofold between the sample sets and (3) a two-sided t test yielded a p value of less than 0.05.

Raw data of all experiments can be accessed at http://www.staff.uni-marburg.de/~buchhol3/RevTrans/

The following gene expression profiling experiments of tissues and *in vivo/in vitro* models for serial characterization of candidate genes were conducted:

#### Primary human tissues

A total of 16 clinical samples from ductal adenocarcinoma, 6 samples from chronic pancreatitis, and 4 samples from morphologically normal resection margins from chronic pancreatitis resectates were analyzed. Samples were provided by the surgery department at the University of Ulm (Germany) as well as the pathology department of the University of Kiel (Germany). Informed consent in writing was obtained from all patients prior to using tissue samples. The study was approved by the local ethics committees at the Universities of Ulm and Kiel (Ethikkommission der Universität Ulm; Ethikkommission der Christian-Albrechts-Universität zu Kiel).

#### Pancreas Development

Total RNA from normal adult human pancreas and fetal human pancreas were purchased from Clontech. Each pooled sample was independently labeled and hybridized 3 times.

#### ATRA treatment of NB4 leukemia cells

The acute promyelocytic leukemia-derived cell line, NB4 [26], was grown at 37°C in 5% CO2 in an RPMI medium supplemented with 2 mM L-glutamine and 10% decomplemented fetal calf serum. Cells were cultured for 48 h with or without 1 μM All-trans retinoic acid (ATRA; Sigma-Aldrich). Duplicates of 3 independent experiments were analyzed.

#### SPC treatment of PaTu II cells

Sphingosylphosphorylcholine (SPC) is a bioactive lipid with multiple biological roles including the regulation of differentiation in embryonic and cancer cells. PaTu II pancreatic cancer cells were maintained in DMEM (Gibco, Invitrogen, Carlsbad, CA) supplemented with 10% (v/v) fetal bovine serum (PAA, Pasching, Austria) in a humidified atmosphere and 5% CO2, 95% air at 37°C. Cells were incubated with 15 μM SPC in serum-free Medium fpr 2 h or left untreated. Duplicates of 3 independent experiments were analyzed.

#### 2D vs. 3D cell cultures

The pancreatic cancer cell line A818-1 was grown either in 2D monolayers or in 3-dimensional “hollow spheres” as described [27]. Total RNA was extracted from 3 independent experiments and analyzed in duplicates.

#### PaTu-8988s and-t differentiation model

PaTu-8988s and PaTu-8988t are two cell lines which were derived from the same liver metastasis of a human primary pancreatic adenocarcinoma, but differ in their differentiation status and metastatic capacity [28]. The poorly differentiated cell line PaTu-8988t was transfected with an E-Cadherin expression construct or vector control, respectively, and individual clones analyzed by expression profiling. In addition, 3 independent preparations of wild-type PaTu-8988s cells were analyzed.

#### SUIT2 metastasis model

S2-007 and S2-028 are cell lines derived from the same primary pancreatic adenocarcinoma, but displying strong differences in their invasive and metastatic potential both in vitro and in vivo [29]. Three independent preparations each of the highly metastatic S2-007 and the low metastatic S2-028 cell line grown in 2D cell culture (DMEM (Gibco, Invitrogen, Carlsbad, CA) supplemented with 10% (v/v) fetal bovine serum (PAA, Pasching, Austria) in a humidified atmosphere and 5% CO2, 95% air at 37°C) were analyzed by expression profiling.

#### Apoptosis-resistance in Capan-1 cells

Pancreatic cancer cells are inherently highly resistant to spontaneous or chemotherapy-induced apoptosis. The human pancreatic tumor cell line Capan-1 (pRB+/p16-) was stably transfected with p16 expression constructs or control plasmids as described [30] to functionally inactivate pRB. Three independent experiments were conducted and analyzed in duplicate.

#### Influence of RAS mutants on the TGFB1-induced phenotype of PANC-1 cells

We have previously described expression profiling of the influence of RAS mutants on the TGFB1-induced phenotype of the pancreatic cancer cell line PANC-1 [24]. In brief, PANC-1 cells stably transfected with a dominant negative HRAS(S17N) mutant, a constitutively active KRAS2(G12V) mutant, or mock transfected with an EGFP expression construct were treated with TGFB1 or left untreated, respectively. Three independent experiments each were analyzed in duplicate.

### Cell lines for parallelized and individual functional assays

Panc-1 [31] and HEK-293 [32] cells were obtained from the American Type Culture Collection (Manassas, USA). S2-007 and S2-028 were from T. Iwamura [33] (Miyazaki Medical College, Miyazaki, Japan). IMIM-PC1 cells were kindly provided by F.X. Real [34] (CNIO, Madrid, Spain).

Cells were cultured in Dulbecco’s modified eagle medium (DMEM) containing 10% FBS and 0.05 mg/ml Gentamicin at 37°C and 5% (v/v) CO2.

### Reverse transfection arrays

Full length open reading frames (ORFs) of all candidate and control genes were purchased as cDNA clones from Open Biosystems (Lafayette, CO, USA), PCR-amplified using specific primers and cloned into the pENTR vector (Invitrogen/Life Technologies, Darmstadt, Germany). ORFs were then shuttled into the pdECFP and pdEYFP expression vectors [35] using the Gateway cloning system (Invitrogen/Life Technologies, Darmstadt, Germany). Each ORF was therefore available as an N-terminal fusion construct with cyan fluorescent protein (CFP) as well as a C-terminal fusion construct with yellow fluorescent protein (YFP).

For production of ‘reverse transfection microarrays’, expression constructs were spotted onto glass slides according to a protocol modified from [22]. 4 μg of each plasmid DNA was mixed with 11 μl PBS and 2 μl Lipofectamine 2000 (Invitrogen, Karlsruhe, Germany) in a 96-well cell culture plate. After 20 min incubation at RT, 15 μl gelatine (0.1% in PBS) was added to each well. Transfection mixtures were arrayed on Superfrost Plus glass slides (Menzel GmbH, Braunschweig, Germany) using a Tecan Miniprep 75 automated sample processor (Tecan, Männedorf, Switzerland), generating spot sizes of 0.5–1.5 mm diameter. Slides were dried and stored up to 3 weeks in a dry atmosphere at 4°C. For generation of live cell microarrays, slides were placed in quadriPERM plates (Sarstedt, Nümbrecht, Germany), seeded with 4.5 x 10^5^ HEK-293, 4 x 10^5^ Panc-1, or S2-007: 5 x 10^4^ S2-007 in DMEM/10% FCS and incubated for 48 h at 37°C.

### Immunocytochemistry

Live cell microarrays were fixed for 15–30 min at room temperature with 4% paraformaldehyde solution in PBS and counterstained and mounted using DAPI-containing mounting medium (Vector Laboratories, Burlingame, USA). Expression of fusion constructs and subcellular localization of the gene products were documented using a Zeiss Axiovert 200 fluorescence microscope (Carl Zeiss GmbH, Göttingen, Germany). Where applicable, optical sections of fluorescent samples were produced at 630x magnification using the Zeiss ApoTome technology (http://www.zeiss.com/microscopy/en_de/products/imaging-systems/apotome-2-for-biology.html).

Antibody incubation and detection were carried out according to the manufacturer’s instructions for the individual antibodies, respectively. The following antibodies were used:

anti-Ki67: 1:400, polyclonal rabbit, ab833, Abcam, Cambridge, UK
anti-Cyclin B1: 1:100, monoclonal mouse, cat. #4135, Cell Signaling Technology, Danvers, USA
anti-ACTIVE-Casapse-3: 1:150, polyclonal rabbit, cat. #G7481, Promega, Madison, USA
anti-Vimentin: 1:200, monoclonal mouse, ab28028, Abcam, Cambridge, UK
anti-E-Cadherin: 1:100, monoclonal mouse, cat. #610181, BD Biosciences, Sparks, USA
anti-rabbit IgG (Cy3) 1:500, polyclonal goat, ab6939, Abcam, Cambridge, UK
anti-mouse IgG (Cy3) 1:500, polyclonal goat, ab97035, Abcam, Cambridge, UK

### mRNA isolation and protein extraction

RNA isolation was carried out using the peqGOLD Total RNA Kit (peqlab Biotechnology GmbH, Erlangen, Germany) according to the manufacturer’s instructions. Concentrations were quantified with the NanoDrop ND-1000 (peqlab Biotechnology GmbH) followed by synthesis of first-strand cDNA from 1 μg total RNA using the Omniscript Kit and protocol (Qiagen, Hilden, Germany).

For total protein extracts, cells were centrifuged in culture medium (13 000 rpm, RT, 5 min). Pellets were resuspended in PBS, supplemented with proteinase inhibitor (G-Biosciences, Maryland Heights, USA) and sonicated with a Labsonic U (B.Braun, Melsungen, Germany). Protein concentrations were determined using Bradford protein assay and measured with the Multiskan FC photometer (Thermo Scientific, Langenselbold, Germany).

### qRT-PCR and immunoblotting

First-strand cDNAs were amplified in 7500 Fast Real-Time PCR System (Applied Biosystems, Warrington, USA) using Power SYBR Green Master Mix (Applied Biosystems) and specific primer pairs designed with the PrimerExpress program (Applied Biosystems, for sequences see supplementary material).

For Western blot, 10 ng of total protein extracts were loaded and separated by 10 or 15% SDS-PAGE. Separated proteins were transferred onto a nitrocellulose membrane (Whatman GmbH, Dassel, Germany) and blocked for 4–6 h at 4°C in 1xTBS, 0.1% Tween 20 and 5% powdered milk. Primary antibodies were diluted 1:1 000 in blocking buffer and incubated overnight at 4°C. TBS-T (0.1%) was used as washing buffer. The HRP-conjugated goat-anti-rabbit secondary antibody was diluted to 1:10 000 and incubated for 1–2 h at 4°C in blocking buffer. Proteins were detected using the ECL immunoblot kit (GE Healthcare Europe GmbH, Freiburg, Germany).

### Construction and analysis of tissue microarrays

For the construction of tissue microarrays (TMAs), 1 mm (tumor) and 1.5 mm (normal tissue) sized tissue biopsies were extracted from paraffin donor blocks and transferred into pre-punched holes on recipient paraffin blocks with a tissue microarrayer (Beecher Instruments, Inc., Sun Prairie, USA) equipped with a TMA booster (Alphelys, Plaisir, France). Grid layouts for tumor and normal pancreatic tissue TMAs were designed with the TMA Designer 2 software (Alphelys). The recipient blocks were sealed for 10 min at 56°C and 30 min at 4°C. This procedure was repeated twice. The TMA blocks were cut into 3.5 μm sections and placed on SuperFrost Plus slides for immunohistochemical staining. The use of human tissues for research purpose was approved by local ethics committee at the University Hospital, Tübingen (470/201BO1), and Clinic of Surgery, University Heidelberg, Germany (301/2001).

Immunohistochemical staining was performed with a mouse monoclonal anti-ADRBK1 antibody (Thermo Fischer, #MA5-15840; 1:1000 dilution) on the Ventana BenchMark XT system (Ventana Medical Systems, Tucson, USA) including the deparaffinization of the formalin-fixed paraffin embedded tissue sections and the heat-induced antigen-retrieval.

Analysis of staining intensities was performed by a pathologists with expertise in pancreatic tumors (B S) in a blinded fashion regarding tumor parameters. The proportion of the positive cells showing a significant staining in tumor areas was estimated in percent and divided into scores (1–10%-weak, 11–50%-moderate, > 50%-strong.

### Transfection of siRNAs

For RNAi-mediated silencing of ADRBK1 expression, pre-designed Silencer siRNAs (Applied Biosystems/Ambion, Austin, TX, USA) were transfected in a concentration of 40 nmol using the siLentFect Lipid Reagent (Bio-Rad, München, Germany). Accell non-targeting siRNA#1 (Thermo Fisher Scientific, Dharmacon Products, Lafayette, LA, USA) was employed as non-silencing control.

### Proliferation and viability assays

For BrdU incorporation assays using the Cell Proliferation ELISA Kit (Roche Diagnostics, Mannheim, Germany), 5 000 to 7 500 cells were reseeded 24 h post-transfection into a ViewPlate-96 Black cell culture plate (Perkin Elmer, Waltham, MA, USA). Following over-night culturing, cells were incubated 4–6 h with BrdU containing medium. After fixation for 1 h, cells were stained for 1.5 h with the peroxidase-conjugated anti-BrdU antibody, chemiluminescence substrate added and the emitted light quantified with a Centro LB 960 luminometer (Berthold Technologies GmbH, Stuttgart, Germany).

For MTT assays, 20 000 to 25 000 cells were reseeded 24 h after transfection in 24-well cell culture plates. After additional culturing for 24 h or 48 h, supernatant was replaced by MTT-containing medium (thiazolyl blue, Carl Roth GmbH) and plates were incubated for 2 h at 37°C. Medium was replaced by solubilization solution (10% Triton-100, 0.1 M hydrochloric acid in 2-propanol). Extinction was measured at 570 nm with the Multiskan FC photometer (Thermo Scientific, Schwerte, Germany).

### Flow cytometry

Trypsinized cells were centrifuged at 1,200 rpm for 3 min. After washing with PBS, pellets were thoroughly resuspended in 50–100 μl PBS and the cell suspension transferred drop-wise into ice-cold ethanol (70%) under continuous vortexing. Following centrifugation at 1 000 g for 5 min, the supernatant was carefully discarded, the pellet washed with 500 μl ice-cold PBS and resuspended in 500 μl of propidium-iodide mixture (1 mg/ml Propidium-iodide (Sigma Aldrich) + 500 μg/ml DNAse-free RNAse (Roche Diagnostics) in PBS). Samples were incubated for 30–45 min at RT in the dark and subsequently measured with the flowcytometer LSR II (BD Biosciences, Heidelberg, Germany). For data evaluation, the software ModFit LT (Verity Software House, Topsham, USA) was used.

## RESULTS

### Serial characterization of PDAC candidate genes by expression profiling using cDNA array hybridizations, data analysis and selection of candidate genes

As a first step in the systematic identification of novel functionally relevant candidate genes in pancreatic cancer, we produced custom cDNA arrays with pancreatic cancer-specific cDNA clone collections (n = 468 from [7] and [36]) which were additionally augmented with large-scale collections of cDNA clones (n = 1492) from potentially relevant gene families such as kinases, phosphatases, serum response genes etc. (see data sets in supplementary information for details of the array compositions). These arrays were then used for gene expression analyses of primary human pancreatic tissues as well as a number of *in vivo* and *in vitro* models of pancreas development, cell differentiation, invasion, metastasis, apoptosis resistance etc. (note that since the cDNA clone collections for the generation of the arrays was continually expanded over the course of these studies, not all cDNAs were hybridized with all experimental models). In order to be selected for further functional characterization, candidate genes had to be significantly deregulated in pancreatic cancer (overexpressed or downregulated in primary pancreatic cancer tissues as compared to chronic pancreatitis and normal pancreas samples), as well as show significant regulation in at least one of the functional model systems (see Material&Methods section for details of hybridization signal quantification and definition of differential expression).

This raw list of potential candidate genes was manually curated to exclude genes which had previously been intensively studied in pancreatic cancer, as well as exclude expressed sequence tags (ESTs) from the pancreatic cancer-specific cDNA collection which showed insufficient homology to annotated human genes. Using these criteria, a total of 50 genes were selected for parallelized functional characterization (see below). This list was augmented with 17 candidate genes which had shown PanIN-specific expression patterns in our previous analyses of microdissected samples of normal ducts as well as pre-cancerous and cancerous lesions from human pancreata [11]. Finally, a selection of 14 known genes with diverse cellular functions were added as positive controls for the functional analyses. The complete list of selected candidate and control genes is given in S1 Table (supplementary information).

### Parallelized cell-based assays

In order to rapidly screen for functional effects of all 67 candidate genes in parallel, expression constructs of the candidate genes as well as 14 control genes with known functions were cloned in the form of N-terminal fusions with cyan fluorescent protein (CFP) as well as C-terminal fusions with yellow fluorescent protein (YFP). The plasmid DNA, complexed with transfection reagents, was arrayed onto standard microscopy glass slides and overlaid with suspensions of pancreatic cancer cells (Panc-1, S2-007) or non-transformed control cells (HEK-293). Under a fluorescence microscope, spots of transfected cells were easily identified due to the specific fluorescent signals of the CFP or YFP fusion proteins, which also allowed direct comparison with neighbouring non-transfected cells.

**Fig 2:**
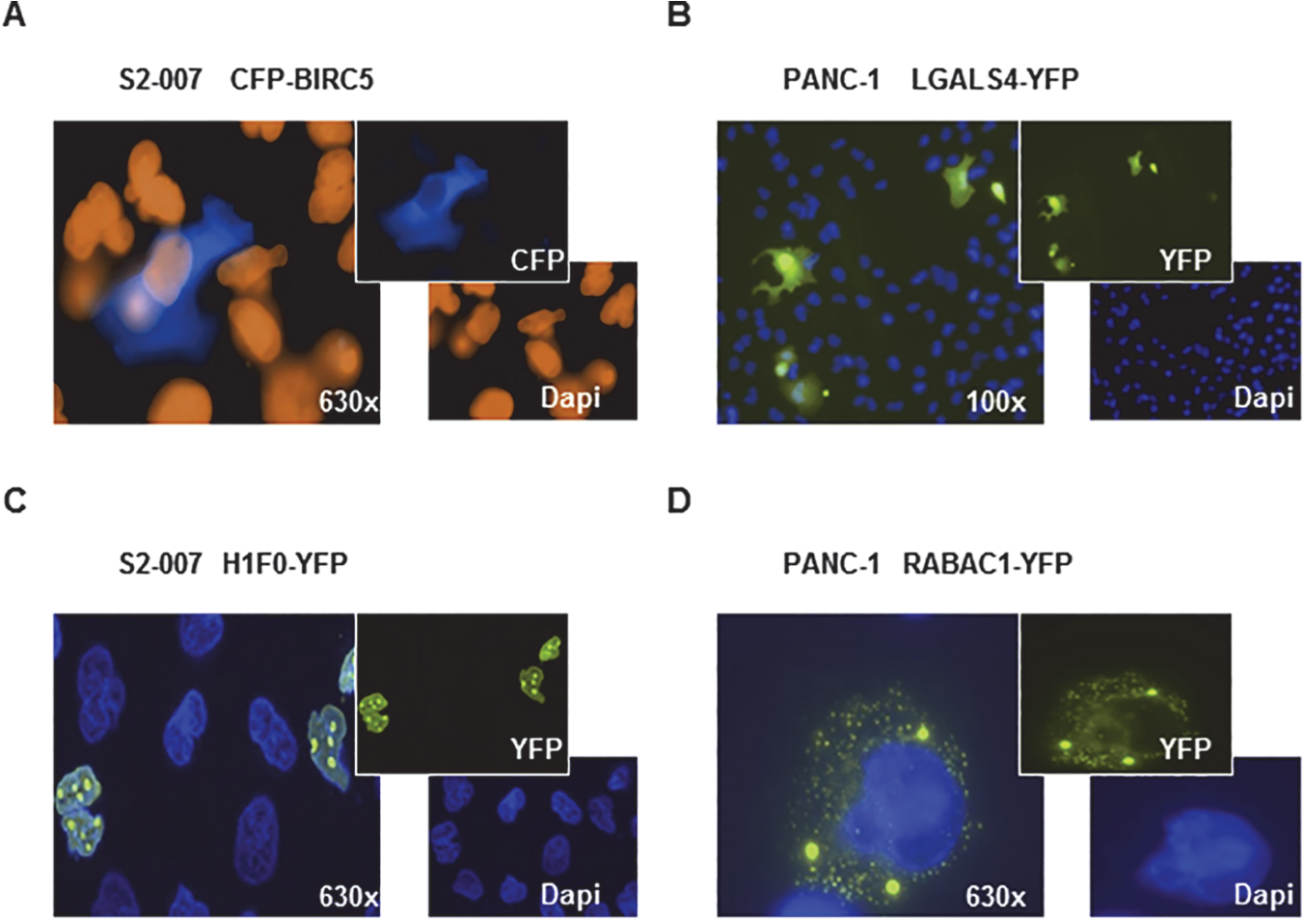
Examples of distinct subcellular localization of fluorescence-tagged candidate gene peoducts after reverse transfection. Shown are CFP (A) or YFP (B-C) fusion constructs. Following transfection and fixation of cells on reverse transfection microarrays, coverslips were applied using DAPI-containing mounting medium. For CFP fusion proteins, DAPI signals are shown in brown (false color) to provide suitable contrast (A). Shown are examples of purely cytoplasmic (A), uniform nuclear-cytoplasmic (B) and purely nuclear localization (C) as well as localization to endosome-like structures (D). Original magnifications are indicated in the images.

**Fig 3:**
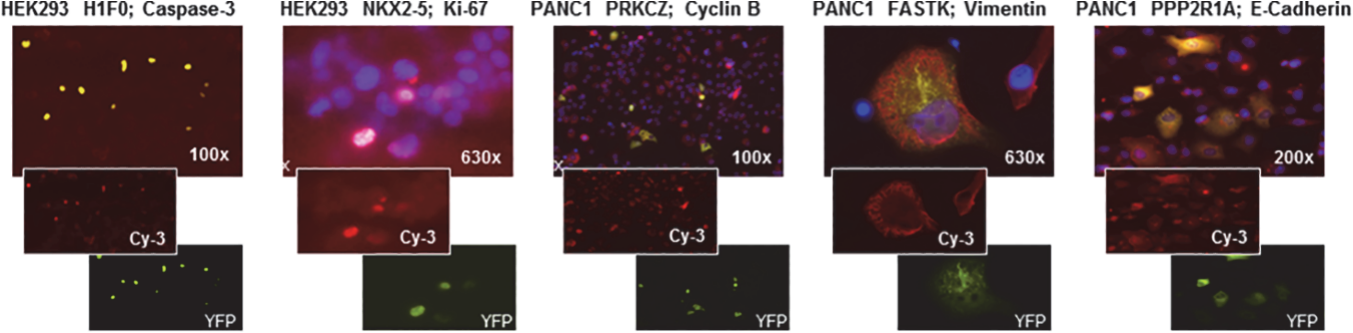
Examples of the different antibody stainings for functional analyses. A: Caspase-3 staining (Cy-3, red), HEK-293 cells transfected with H1F0-YFP (green); B: Ki-67 staining (Cy-3, red), HEK-293 transfected with NKX2-5-YFP (green); C: Cyclin B staining (Cy-3, red), PANC-1 transfected with PRKCZ-YFP (green); D: Vimentin staining (Cy-3, red), PANC-1 transfected with FASTK-YFP (green); E: E-Cadherin staining (Cy-3, red), PANC-1 transfected with PPP2R1A-YFP (green). Original magnifications are indicated in the images.

The reverse transfection arrays were then utilized for parallelized functional analyses in order to identify novel candidate genes with potentially important functions in pancreatic cancer cells. In a first round of evaluation, subcellular localization of the gene products (as well as overall morphology compared to non-transfected cells), both in the presence and absence of serum, was recorded and compared between cancer and control cells (see Fig 2 for examples of different localizations). In subsequent analyses, arrays of fixed cells were incubated with antibodies against well-defined markers of apoptosis (cleaved caspase-3), proliferation (Ki-67, Cyclin B) and epithelial-mesenchymal transdifferentiation (EMT; mesenchymal marker vimentin, epithelial marker E-cadherin). Examples of staining results are displayed in Fig 3. Frequency and intensity of staining were recorded, visually scored and compared between transfected and non-transfected cells. Recurring differences between transfected and non-transfected cells were graded as “++” (strong effects), “+” (moderate but reproducible effects) and “(+)” (weak and/or variable effects). Table 1 lists all genes for which at least one score of “++” or at least three scores of “+” were recorded in independent replicate experiments and which were thus defined as suitable for individual in-depth analyses. The selected candidates comprised 3 kinases (ADRBK1, FASTK, PRKCZ), 3 phosphatases (PPM1G, PPP2R1A, PPP2R4), 2 transcription factors (FRA-2, GTF2F2), 2 actin-interacting proteins (CFL1, RRAS), 1 transmembrane protein (TM4SF1), 1 histone methyl transferase (SUV39H1), 1 G protein-coupled receptor (EBI2) and 1 interferone-inducible protein with unknown cellular function (IFI27). None of them had been associated with pancreatic cancer at the time they were included in the reverse transfection arrays.

**Table 1:** List of genes showing reproducible effects in parallelized assays.

		**localization**			**apoptosis**				**proliferation**				**EMT**		
		**HEK293 vs. transf.**	**10% FCS vs. 0% FCS**	**CFP vs. YFP**	**Caspase 3 + 10% FCS**		**Caspase 3 + 0% FCS**		**Ki67**		**CyclinB1**		**Vimentin**		**E-Cadherin**
**Gene**	**tissue expression**				**Hek293**	**Panc1**	**Hek293**	**Panc1**	**Hek293**	**Panc1**	**Hek293**	**Panc1**	**Hek293**	**Panc1**	**Panc1**
**ADRBK1**	**Up**	** **	**++**	** **	**+**	** **	** **	** **	** **	** **	** **	** **	** **	** **	** **
**CFL1**	**Up**	** **	** **	** **	** **	** **	** **	**+**	** **	**++**	** **	**(+)**	** **	**(+)**	** **
**FASTK**	**Up**	** **	** **	**+**	** **	**+**	** **	** **	**+**	**++**	** **	** **	** **	** **	** **
**PPM1G**	**Up**	**+**	**++**	** **	**+**	** **	** **	** **	** **	** **	** **	** **	** **	** **	** **
**PPP2R1A**	**Up**	**+**	** **	** **	** **	** **	** **	** **	** **	** **	** **	**+**	** **	** **	**++**
**PPP2R4**	**Up**	** **	**+**	** **	** **	** **	** **	**+**	**+**	**+**	** **	** **	** **	** **	**++**
**TM4SF1**	**Up**	** **	** **	** **	** **	**+**	** **	** **	**+**	**+**	** **	** **	** **	** **	**++**
**SUV39H1**	**Up**	** **	**+**	**+**	**++**	**++**	** **	** **	** **	** **	** **	** **	** **	** **	** **
**PRKCZ**	**Up**	** **	** **	** **	**+**	** **	** **	**++**	** **	** **	** **	** **	** **	** **	** **
**RRAS**	**Up**	**+**	**+**	** **	** **	** **	** **	** **	** **	** **	** **	** **	** **	** **	** **
**EBI2**	**Down**	** **	** **	**++**	** **	** **	** **	** **	** **	** **	** **	** **	** **	** **	** **
**FRA-2**	**Down**	**++**	** **	** **	**+**	**++**	**+**	**++**	** **	** **	** **	** **	**(+)**	** **	** **
**GTF2F2**	**Down**	**++**	** **	** **	**+**	**+**	** **	**+**	** **	** **	** **	** **	** **	** **	** **
**IFI27**	**PanIN**	** **	** **	**++**	**+**	** **	** **	**+**	** **	** **	** **	** **	** **	** **	**+**

Recurring differences between transfected and non-transfected cells were graded as “++” (strong effects), “+” (moderate but reproducible effects) and “(+)” (weak and/or variable effects). Expression of the genes as determined by microarray analyses of primary human tissues is indicated. “Up”: genes overexpressed in human PDAC; “Down”: genes downregulated in human PDAC; “PanIN”: genes selected from the analyses of microdissected PanIN lesions.

### Functional validation / in-depth individual characterization of candidate gene ADRBK1

As a proof-of-concept to confirm the validity of functional data obtained via the parallelized cell-based assays, we exemplarily show detailed *in vitro* functional characterization data of one gene, ADRBK1, which was the first gene (in alphabetical order) on the list of high-priority candidates (Table 1). Detailed *in vivo* and *in vitro* characterization of other candidate genes up to the generation of genetic engineered mouse models are either in press or in preparation and will be reported elsewhere.

#### Subcellular localization of the ADRBK1/CRK2 protein

**Fig 4:**
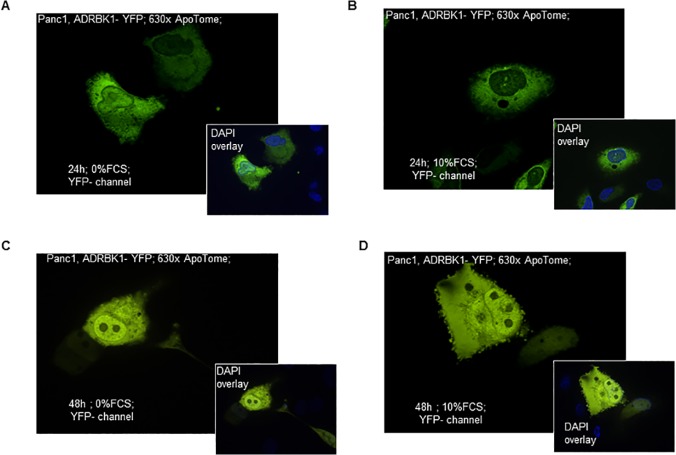
Validation of growth condition-associated subcellular localization of ADRBK1. ADRBK1-YFP fusion constructs (green signals) were transfected into cultured Panc-1 cells cultured in the absence (A, C) or presence (B, D) of 10% serum. After 24h or 48h of incubation, cells were fixed and counterstained with DAPI (blue signals). After 24h, ADRBK1-YFP signals were detectable in both, cytoplasm and nuclei, of cells cultured without serum (A), while nuclei of cells cultured with serum were devoid of YFP fluorescence (B). In contrast, after 48h of incubation, cells grown under either condition showed prominent staining of nuclei (C, D). Optical sections of fluorescent images were obtained using the ApoTome technology. Original magnifications are indicated in the images.

ADRBK1 (Adrenergic, Beta, Receptor Kinase 1) also known as GRK2 (G-Protein Coupled Receptor Kinase 2) was originally included in the cDNA arrays as a member of the kinase gene family, which represent ideal druggable target genes. It was found to be overexpressed in PDAC during the serial expression profiling experiments. In the reverse transfection array experiments, ADRBK1/CRK2 had shown marked differences in subcellular localization depending on the presence or absence of serum (cytoplasmic localization in the presence, nuclear and cytoplasmic localization in the absence of serum). Traditional (“forward”) transfection of ADRBK1 fusion constructs in Panc1 cells confirmed this observation, showing the expected fluorescent signals in the nucleus exclusively in serum-deprived cells 24h after transfection (Fig 4A and 4B). Interestingly, after prolonged incubation times, there was a strong tendency of the signal to shift towards the nucleus regardless of the presence or absence of serum (Fig 4C and 4D), possibly indicating a role of ADRBK1 in the cells’ reaction to the depletion of nutrients and/or growth factors.

#### ADRBK1 overexpression promotes growth in human PDAC

**Fig 5:**
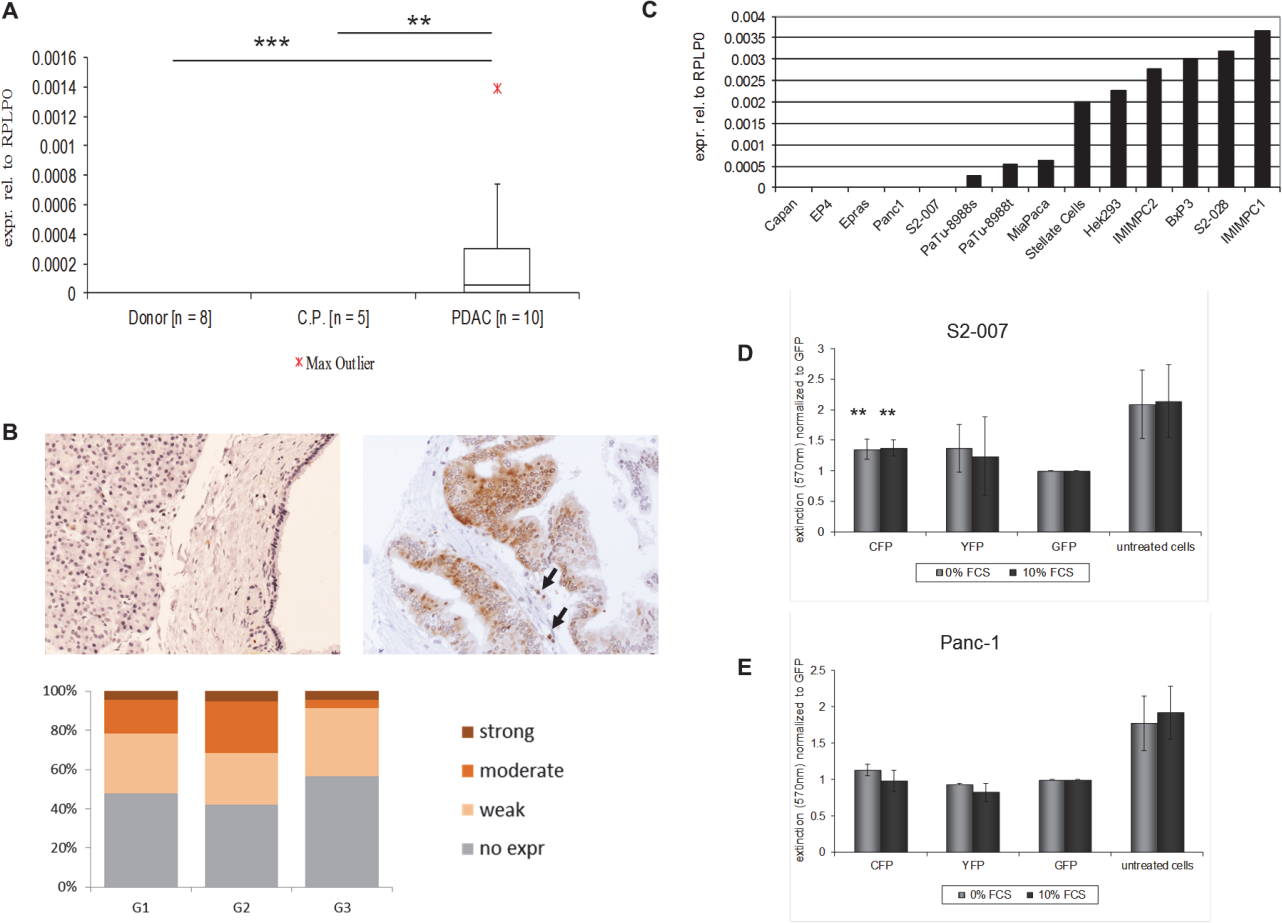
ADRBK1 overexpression promotes growth in human PDAC. A: Box-and-whisker plot showing ADRBK1 mRNA expression in primary human pancreatic tumor tissue samples, chronic pancreatitis and normal pancreas as analyzed by quantitative realtime reverse transcription PCR (qRT-PCR). Expression was normalized to ribosomal protein, large, P0 (RPLP0) mRNA levels. Data in Fig. represent median and 2nd and 3rd quartiles (boxes) as well as minimum and maximum values (whiskers). CP = Chronic Pancreatitis. ** p<0.01, *** p<0.001 (Student’s t-test). B: Immunohistological analyses of ADRBK1 expression using human pancreatic tissue microarrays (TMAs) comprising 33 donor, 28 chronic pancreatitis and 65 tumor tissues. Micrographs are representative of typical staining results for donor samples (left panel), which were devoid of ADRBK1 staining, or ADRBK1-positive neoplastic tissues (right panel) (original magnification 100x). Note that in positive PDAC cases, neoplastic ducts as well as individual infiltrating immune cells (arrows) stained postitve for ADRBK1. Lower panel: Distribution of staining intensities across different tumor grades (G1: n = 23; G2: n = 19; G3: n = 23). C: TTK mRNA expression levels in a variety of different pancreatic cancer and control cell lines (qRT-PCR; expression was normalized to ribosomal protein, large, P0 (RPLP0) mRNA levels). D, E: MTT assays demonstrated increased numbers of viable S2-007 (C) and Panc-1 (D) cells 72h after transfection of fluorescence-tagged ADRBK1 expression constructs (CFP: N-terminal CFP-ADRBK1 fusion; YFP: C-terminal ADRBK1-YFP fusion) compared to GFP controls in the presence (dark bars) or absence (light bars) of 10% serum in the culture medium.

In order to confirm that ADRBK1 is overexpressed in human pancreatic cancer, primary human tissues were analyzed for ADRBK1 expression by quantitative RealTime PCR. ADRBK1 mRNA levels were undetectable in all normal pancreas samples analyzed (n = 8), as well as in 4 of 5 chronic pancreatitis samples, whereas strong expression was detected in 5 out of 10 pancreatic cancer tissues (Fig 5A). More detailed analyses on the protein level corroborated these results. Immunohistochemical staining of tissue microarrays (TMAs) comprising 33 normal pancreas samples, 28 samples of chronic pancreatitis tissues and 65 PDAC samples (grades 1–3) cornfirmed that ADRBK1 protein was undetectable in all non-neoplastic tissues (chronic pancreatitis and normal pancreas), whereas ADRBK1 staining was readily detectable in 33 out of 65 (51%) PDAC samples (Fig 5B). Interestingly, ADRBK1 signal in positive samples was detectable in both, epithelial cancer cells as well as subsets of infiltrating immune cells (Fig 5B, upper right panel). Staining frequency and/or intensity did not vary systematicall with tumor grade (Fig 5B, lower panel).

Analysis of ADRBK1 mRNA levels in a broad panel of transformed as well as non-transformed cell lines revealed profound differences in ADRBK1 expression. Of interest, Panc-1 and S2-007 cells, which had been used for the parallelized functional assays with recombinant expression of fluorescence-tagged candidate genes, showed no detectable endogeneous ADRBK1 expression, while the majority of pancreatic cancer cell lines expressed moderate or high levels of ADRBK1 mRNA (Fig 5C). In order to assess the functional consequences of recombinant ADRBK1 expression in the negative cell lines, S2-007 and Panc-1 cells were transiently transfected with CFP-ADRBK1 or ADRBK1-YFP expression constructs or GFP as a negative control, respectively. MTT cell viability assays performed 72h after transfection demonstrated a trend to increased proliferation in the ADRBK1-expressing cells compared to GFP controls, although statistical significance was only reached in the case of S2-007 cells transfected with the CFP-ADRBK1 construct (Fig 5D and 5E).

#### Inhibition of endogeneous ADRBK1 expression strongly impairs cell growth

**Fig 6:**
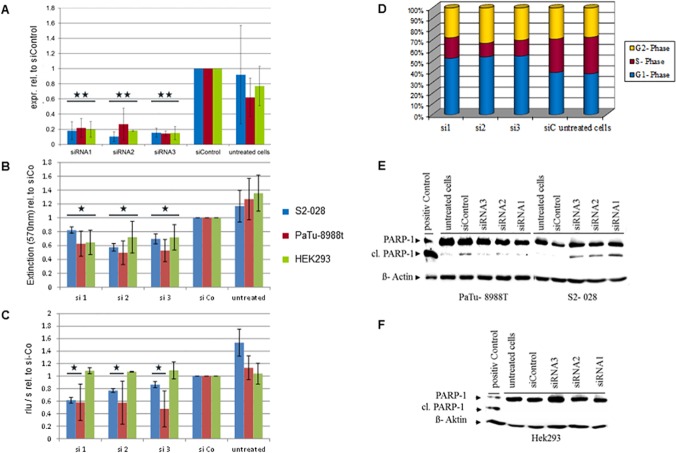
Inhibition of endogeneous ADRBK1 expression impairs cell growth. A: Transfection of three individual siRNAs against ADRBK1 resulted in at least 70% reduction of ADRBK1 mRNA levels in two pancreatic cancer cell lines with high (S2-28) and intermediate (PaTu-8988t) levels of endogeneous ADRBK1 expression, as well as the non-transformed HEK293 cell line. mRNA levels were determined by qRT-PCR and normalized to non-silencing control siRNA (“siControl”). B: MTT assays showed significantly reduced numbers of viable cells 72h after transfection of ADRBK1-specific siRNAs as compared to non-silencing control siRNA. C: PI-staining and flow cytometry analyses were performed 48h after siRNA transfection into PaTu 8988t cells. The results demonstrate strongly increased proportions of cells in G1 phase and strongly decreased proportions of cells in S phase after ADRBK1 knockdown, while G2 phase remained essentially unchanged. Shown is one representative example of three independent experiments.E, F: PARP cleavage was analyzed by Western Blot analyses 72h after transfection of ADRBK1-specific and control siRNAs, respectively. The results showed slightly elevated levels of cleaved PARP protein in S2-028 cells (E, right panel), but this was not apparent in PaRu-8988t (E, left panel) or HEK293 (F) cells. * p<0.05, ** p<0.01 (Student’s t-test).

To more thoroughly study the role of endogeneous ADRBK1 in pancreatic cancer cells, two cell lines with moderate (PaTu 8988t) and high (S2-028) mRNA expression were subjected to siRNA-mediated transient inhibition of ADRBK1 expression. As an additional reference, immortalized but non-transformed HEK293 cells, which also show strong ADRBK1 mRNA expression, were likewise examined. siRNA transfection routinely resulted in ~80% reduction in ADRBK1 mRNA levels in all three cell lines and with all three independent siRNAs (Fig 6A). While MTT assays uniformly demonstrated a clear and statistically significant reduction in the numbers of viable cells for all three cell lines 72h after transfection of any of the three siRNAs (Fig 6B), BrdU assays, which were performed 48h post transfection and directly assessed DNA synthesis rates as a measure of cell proliferation, showed a more differentiated picture. PaTu 8988t cells, which have a lower level of endogeneous ADRBK1 expression than S2-028 or HEK293 cells, were most strongly affected by the knockdown, resulting in more than 40% reduction in proliferation activity for all three siRNAs compared to cells transfected with non-silencing control siRNA (Fig 6C, red bars). S2-028 cells showed an intermediate phenotype, with varying but statistically significant reduction of proliferation rates for the individual siRNAS (Fig 6C, blue bars), while HEK293 cells were completely unaffected by the treatment (Fig 6C, green bars). Flow cytometry analyses of PaTu8988t cells with and without ADRBK1 knockdown confirmed the strong impairment of proliferative activity, demonstrating a dramatic reduction of the proportion of cells in S-phase and a strong increase of cells in G1 suggestive of a G1/S cell cycle arrest (Fig 6D).

In order to assess whether inhibition of ADRBK1 expression also induces apoptosis in the cells, we performed Western Blot analyses in ADRBK1 knockdown and control cells. While ADRBK1 knockdown resulted in slightly elevated levels of PARP-1 cleavage, an indicator of apoptosis, in S2-028 cells (Fig 6E, right panel), levels of cleaved PARP-1 protein were not appreciably elevated above control levels in PaTu 8988t cells (Fig 6E, left panel), and were completely absent in HEK293 cells (Fig 6F). Analysis of caspase-3 cleavage showed similar results (data not shown), indicating that apoptosis induction is not a major downstream effect of ADRBK1 inhibition in PDAC cell lines.

## DISCUSSION

Cancers are polygenic diseases arising from the accumulation of multiple genetic and epigenetic defects in the affected cells. High-throughput and high-content screening technologies, such as DNA microarrays and next-generation sequencing, have enabled the rapid accumulation of comprehensive catalogues of molecular changes that accompany malignant cell transformation. Taking pancreatic cancer as an example, more than 360 differentially expressed genes were detected by our own group in the first ever expression profiling analysis of a solid tumor [7], and many more genes have been added to the list since (for a comprehensive database of gene expression changes in pancreatic cancer, see http://www.pancreasexpression.org/). In principle, this should lay the basis for an improved understanding of the biology of this disease and the development of more effective treatment modalities. However, exploitation of the results is complicated by the fact that many of the observed alterations merely represent epiphenomena of tumorigenesis which themselves don’t actively promote tumor formation or progression.

In order to avoid spending valuable time and resources on the analysis of this type of ancillary alterations, we have implemented our multi-step strategy to systematically select novel candidate genes of functional importance in pancreatic cancer cells. As with many high-content screening approaches, the strength of this strategy lies in the positive selection of functionally relevant genes rather than its “negative predictive value”, i.e. the fact that a gene does not show any positive results in these analyses does not necessarily exclude important functional roles of this gene in tumorigenesis. As a first important limiter, the reverse transfection array analyses as implemented here can, by design, only detect gene functions related to cell-autonomous mechanisms and effects. In contrast, it is well established that numerous interactions of pancreatic cancer cells with different cell types of the surrounding inflammatory stroma (e.g. pancreatic stelate cells (PSC) and tumor-associated macrophages (TAM)) profoundly influence the onset and progression of pancreatic tumors and may directly affect proliferation, survival and invasion of tumour cells [37–41]. In addition, not all functional roles and effects may be addressable by the recombinant overexpression format of fluorescently labeled expression constructs as implemented on our reverse transfection arrays.

These limitations notwithstanding, our approach has proven highly successful as demonstrated by the results of the in-depth analyses of novel candidate genes. In total, 14 genes were selected as high priority candidates for individual functional analyses. Notably, 8 of the 14 candidates (3 kinases, 3 phosphatases, 1 histone methyl transferase and 1 G protein coupled receptor) were from gene families that are generally considered to be good targets for drug development, i.e. their enzymatic functions can potentially be very specifically addressed by small molecule inhibitors.

In addition to ADRBK1/GRK2, for which results are presented in this study, important functional roles of the next two genes on the alphabetical list, CFL1 and FASTK, were likewise confirmed and expanded in individual analyses (results will be presented elsewhere). Moreover, pro-tumorigenic functions of our candidate genes SUV39H1 and TM4SF1 have been independently reported in recent publications [42,43]. Functional validation of the remaining candidates on the list are ongoing.

ADRBK1 has originally been described as a ubiquitously expressed kinase that phosphorylates, and thereby regulates, ß-adrenergic receptors [44]. The kinase has later been shown to interact with several G protein-coupled receptors (GPCRs) as well as a growing list of non-receptor substrates, and its role in physiology and pathophysiology of the cardiovascular system has been intensively studied (for reviews, see [45,46]). Putative functions of ADRBK1 in cancer have only recently been addressed in a small number of studies, and a functional role of the kinase in pancreatic cancer has not been described to date. Interestingly, the role of ADRBK1 in different tumors seems to be highly tissue- and cell type-specific. While overexpressed on the protein level in primary non-medullary thyroid cancer tissue as compared to adjacent normal tissue, recombinant overexpression of ADRBK1 in two poorly differentiated thyroid cell lines has been reported to result in significantly decreased proliferation of the cells, suggesting an anti-proliferative role of ADRBK1 in these cells [47]. Similar results have been obtained in hepatocellular cancer (HCC) cell lines, where recombinant overexpression of ADRBK1 likewise resulted in decreased proliferation [48]. No information was provided on expression levels of the kinase in primary HCC tissues, though.

A third study reported indirect effects of endothelial cell-specific ablation of ADRBK1 expression on the growth of xenotransplanted melanoma cells. Tumors grew significantly larger in animals with lower endothelial levels of ADRBK1, presumably due to decreased pericyte coverage of the endothelium and altered recruitment of macrophages in response to ADRBK1 downregulation in the endothelium [49]. It remains unclear, though, if ADRBK1 also has a direct growth-regulatory role in melanoma cells, since expression levels in the xenotransplanted cells were not manipulated.

Finally, expression of an inhibitory peptide derived from the c-terminus of the ADRBK1 protein strongly inhibited growth of prostate cancer cells *in vitro* and *in vivo*, suggesting that GPCR signaling is a central positive regulator of cell cycle progression in these cells [50], although it remains unclear if this effect is directly linked to a specific regulatory function of ADRBK1 or results from global inhibitory effects of the c-terminal fragment.

Our own results clearly demonstrate that ADRBK1 is not only significantly overexpressed in human pancreatic cancer, but also mediates pro-proliferative effects in pancreatic cancer cells. Recombinant overexpression of the kinase in cell lines without endogenous expression accelerated cell growth, while RNAi-mediated knockdown of endogeneous ADRBK1 expression significantly inhibited growth through induction of a G1/S cell cycle arrest. The initial (and subsequently confirmed) observation in the reverse transfection experiments which prompted the selection of ADRBK1 for further in-depth analyses was the growth condition-specific translocation of the fluorescence-tagged protein from the cytoplasm to the nucleus. Specific and well-regulated nuclear translocation events, in turn, may be indicators of direct gene-regulating functions of the involved proteins. A prime example of this is the ‘nuclear factor of activated T-cells 2’ (NFAT2 = NFATc1), whose gene regulatory activity is primarily regulated on the level of subcellular localization and which we have previously shown to be a central governor of cell growth in pancreatic cancer [51]. Further studies will have to be conducted in order to identify the cellular targets of ADRBK1 kinase activity in pancreatic cancer cells and to determine to what extent the nuclear translocation of the protein impacts the gene expression profiles of the cells. In addition, it will be of great interest to investigate a possible role of ADRBK1 in the cross-talk between epithelial cancer cells and infiltrating immune cells in PDAC, since our TMA analyses demonstrated concordant expression in tumor and immune cells in ADRBK1-positive tissues from primary human tumors, whereas isolated expression in immune cells in the absence of positive staining of tumor cells or expression in immune cells from chronic pancreatitis tissues was never observed.

To conclude, we demonstrate here that a multistage strategy based on serial gene expression profiling analyses and serial functional characterisation using reverse transfection arrays provides a rational basis for the selection of highly relevant novel candidate disease genes for pancreatic cancer.

## Supporting Information

S1 Table List of genes represented on the reverse transcription arrays. Raw data of all gene expression profiling experiments can be accessed at http://www.staff.uni-marburg.de/~buchhol3/RevTrans/:
(XLS)

S2 Table Data set on primary tissues. (XLSX)

S3 Table Data set on pancreas development. (XLSX)

S4 Table Data set on ATRA treatment of NB4 cells. (XLSX)

S5 Table Data set on SPC treatmentof PaTuII cells. (XLSX)

S6 Table Data set on 2D vs. 3D culture. (XLSX)

S7 Table Data set on the PaTu-8988s and-t differentiation model. (XLSX)

S8 Table Data set on the SUIT2 metastasis model. (XLSX)

S9 Table Data set on Apoptosis resistance in Capan-1 cells. (XLSX)

S10 Table Data set on the influence of RAS mutants on the TGFB1-induced phenotype of PANC-1 cells. (XLSX)
